# *Cavia porcellus* (Rodentia: Caviidae) as host for *Ctenocephalides felis felis* (Siphonaptera: Pulicidae) in artificially infestation

**DOI:** 10.29374/2527-2179.bjvm003123

**Published:** 2023-12-05

**Authors:** Thalita Xavier de Araujo da Silva, Gabriela Pereira Salça de Almeida, Debora Azevedo Borges, Victor Elias Caceres Rios, Thaís Ribeiro Correia

**Affiliations:** 1 Veterinarian, MSc. Programa de Pós-Graduação em Ciencias Veterinárias (PPGCV), Departamento de Parasitologia Animal (DPA), Instituto de Veterinária (IV), Universidade Federal Rural do Rio de Janeiro (UFRRJ). Seropédica, Seropédica, RJ, Brazil.; 2 Veterinarian, DSc. PPGCV, IV, UFRRJ. Seropédica, Seropédica, RJ, Brazil.; 3 Veterinarian, DSc. DPA, IV, UFRRJ. Seropédica, Seropédica, RJ, Brazil.

**Keywords:** flea, infestations, guinea pig, pulga, infestação, porquinho-da-índia

## Abstract

The number of guinea pigs is increasing as pet and their domestication necessitates the study of their pathology and emerging diseases. This study aimed to evaluate guinea pigs' capacity to be infested by *Ctenocephalides felis felis* fleas, as it is a common parasite among pets that causes irritation, stress, and transmission of other pathogens. Seventeen guinea pigs were infested with *C. felis felis*. After 48 hours, the animals were combed, and the number of fleas was determined. Guinea pigs had a very low recovery (average of 5%), leading us to conclude that they are not good hosts for this species, despite some literature citing it as an accidental host if infested along with dogs or cats.

## Resumo

Os porquinhos-da-índia como animal de estimação têm aumentado cada vez mais, e, sua domesticação trouxe a necessidade de estudos sobre suas patologias e doenças emergentes. Este estudo teve como objetivo avaliar a capacidade de infestação dos porquinhos-da-índia pela pulga *Ctenocephalides felis felis*, ectoparasita comum entre os animais domésticos que causa irritação, estresse e transmissão de outros patógenos. Dezessete animais foram infestados com *C. felis felis*. Após 48 horas, os animais foram penteados e o número de pulgas determinado. Os porquinhos-da-índia tiveram uma recuperação muito baixa (média de 5%), levando-nos a concluir que estes não são bons hospedeiros para essa espécie de pulga, apesar de algumas literaturas os citarem como hospedeiro acidental se conviverem juntamente com cães ou gatos infestados.

Fleas (Insecta, Siphonaptera) are important vectors of pathogens in many parts of the world, and their infestation in pets and home environments is common, becoming a major nuisance pest responsible for producing and transmitting diseases in humans and pets. The main species discussed is the cat flea, *C. felis felis* (Dryden & Rust, 1994). The domestication of guinea pigs has exposed them to new pathogens when in contact with other animals. There are reports about rodents kept along with dogs or cats infested with *Ctenocephalides* spp. may also become infested with fleas (Cubas et al., 2014; Fehr & Koestlinger, 2013). Different treatments of guinea pigs have been tested for mites, lice, and fleas (Eshar & Bdolah-Abram, 2012; Kim et al., 2008; Nath, 2016; Vidal et al., 2006). They have also been used as experimental models to study allergic reactions to the bites of *C. felis felis* fleas (Benjamini et al., 1963). Therefore, to elucidate the interactions between fleas and guinea pigs, this study aimed to evaluate the infestation capacity of guinea pigs by cat fleas.

The study was performed according to protocol number 9459140519, submitted to and approved by the Ethics Committee on Animal Use (CEUA) of the Federal Rural University of Rio de Janeiro’s (UFRRJ) Veterinary Institute (IV). The experiment was performed at the Laboratory of Experimental Chemotherapy in Veterinary Parasitology (LQEPV) of the Department of Animal Parasitology, IV of the UFRRJ, located in the city of Seropédica, Rio de Janeiro, Brazil. Eighteen male and female American Shorthair guinea pigs (*C. porcellus*) were separated into cages individually to investigate the susceptibility of each animal. Cat fleas (*C. felis felis*) were collected from a colony established and maintained in LQEPV and one hundred unfed adult fleas aged 7-14 days old was used to infest each animal. The flea colonies were maintained using a protocol approved by the CEUA of UFRRJ’s IV (091/2014).

The animals were infested and 48 hours later, the fleas were combed out and counted per body area: head and back of the neck (HN), mentonian region (MR), tail (T), left side (LS), right side (RS), and abdomen (AB). This study was adapted from Marchiondo et al. (2013). One animal was excluded from the study due to suspected dermatophytosis. Table 1 shows the number of counted fleas per body region. A very low recovery (an average of 5%; 22% maximum and 0% minimum) was observed in all individuals. The highest prevalence was observed at the base of the tail, followed by the laterals.

**Table 1:** Recovery of *Ctenocephalides felis felis* fleas per body region on artificially infested guinea pigs.

**Data**	**Recovery of fleas per body region on guinea pigs**					
	**HN**	**MR**	**Tail**	**Left side**	**Right side**	**Abdomen**
Min-Máx	0-5*	0-1	0-6	0-5	0-4	0-3
Average	0.76	0.18	1.41	1.06	0.76	0.82

HN: Head and back of the neck; MR: Mentonian Region;

* In number of specimens.

In 2006, Vidal et al. described topical fipronil tested at different doses and times of action in naturally infested guinea pigs, however, the paper did not mention what flea species encountered. Benjamini et al. (1961) and Larrivee et al. (1964) have used guinea pigs as an experimental model to analyze skin reactions to flea bites. The cat fleas were not infested but placed unfed in a shaved area for a few minutes, and then removed, suggesting that these experiments were not conclusive in determining if guinea pigs are good hosts for the cat flea. There have been no other reports of flea infestations in guinea pigs.

The results of this experiment indicate that the artificial infestation of fleas in guinea pigs was inefficient. The data allowed us to deduce that guinea pigs are not good flea experimental models and possibly are not naturally susceptible to *C. felis felis* infestation.
